# Complete Genome Sequence of Type 1 Porcine Reproductive and Respiratory Syndrome Virus Strain E38, Isolated from South Korea with a Novel Deletion

**DOI:** 10.1128/genomeA.01180-15

**Published:** 2015-10-15

**Authors:** Jeong-Min Kim, Young-Woo Kwon, Eun-Jin Choi, In-Ohk Ouh, Se-Eun Choe, Jienny Lee, Jae-Young Song, Sang-Ho Cha

**Affiliations:** Viral Disease Division, Animal and Plant Quarantine Agency, Anyang, Republic of Korea

## Abstract

We report the complete genome sequence of the European type 1 porcine reproductive and respiratory syndrome virus E38 strain, isolated from South Korea with a novel deletion. It contains a 61-nucleotide discontinuous deletion of the Nsp2 and Nsp12 regions. This study will aid in understanding the genetic diversity of type 1 PRRSV and in manufacturing a construct based on Korean vaccine candidate development.

## GENOME ANNOUNCEMENT

Porcine reproductive and respiratory syndrome virus (PRRSV) is a member of the family *Arteriviridae* in the order *Nidovirales* (1, 2). The genome is a small-envelope virus with a positive-sense, single-stranded RNA genome of approximately 15 kb in length and contains a cap structure at its 5′ end and a poly (A) tail at its 3′ end. The genome consists of 5′ and 3′ noncoding regions (NCRs) and at least nine open reading frames (ORFs), including ORF 1a and 1b, ORF 2a and 2b and ORFs 3 to 7 (3, 4). PRRSV is known as the causative agent of a severe reproductive failure in sows and respiratory problems in all ages of groups of pigs and was recognized as one of the most common and economically significant infectious diseases in the pig industry worldwide in the late 1980s (5–7). PRRSV is distinguished genetically and serologically by two distinct genotypes: the European (EU) (type 1) and North American (type 2) types each have prototype Lelystad and VR-2332 strains (8, 9). Both types share an approximately 60% sequence homology (10). In South Korea, the North American–type PRRSV and the EU-type PRRSV were first isolated in 1997 and 2007, respectively (11, 12). After the isolation in 2007, the genetic diversity of the EU-type PRRSV in South Korea (13, 14) was reported. Here, we announce the complete genome sequence of the EU-type PRRSV E38 strain.

The strain, E38, was isolated from a swine farm infected with PRRSV in 2007. Total RNA was extracted from the serum using the RNeasy mini kit (Qiagen, Germany) according to the manufacturer’s instructions. The complete genome of E38 was generated with reverse transcriptase PCR (RT-PCR) using 6 pairs of primers amplifying 6 overlapped fragments. The primers were designed using the CLC Main Workbench version 7.0.3 (Qiagen) based on the sequence of the Lelystad virus. The 6 amplified RT-PCR products were purified and cloned into a pGEM-T Easy vector (Promega) and sent to Macrogen, Inc. for sequencing. The complete genome of E38 comprised 15,065 nucleotides (nt), including the 3′ poly (A) tail. Comparison of E38 with the EU prototype Lelystad virus by multiple alignment showed that there are 61-nt discontinuous deletions: 60-nt deletions in the Nsp2 region (3-nt deletions at positions 1916 to 1918 and 57-nt deletions at positions 2450 to 2506) and a 1-nt deletion in position 11759 of the Nsp12 region. Furthermore, E38 showed 88.83% nucleotide homology with the genome of the Lelystad virus.

Taken together, the complete genome sequence data of E38 indicates that the EU genotype PRRSV variation occurs in South Korea. This information will aid in understanding the genetic diversity of the EU genotype PRRSV in South Korea and in manufacturing an E38 infectious clone construct based on Korean vaccine candidate development.

### Nucleotide sequence accession number.

The complete genome sequence of the PRRSV E38 strain is available in GenBank under the accession number KT033457.
